# Coarse Particles and Heart Rate Variability among Older Adults with Coronary Artery Disease in the Coachella Valley, California

**DOI:** 10.1289/ehp.8856

**Published:** 2006-04-25

**Authors:** Michael J. Lipsett, Feng C. Tsai, Linda Roger, Mary Woo, Bart D. Ostro

**Affiliations:** 1 California Department of Health Services, Environmental Health Investigations Branch, Richmond, California, USA; 2 Department of Epidemiology and Biostatistics, School of Medicine, University of California, San Francisco, California, USA; 3 California Office of Environmental Health Hazard Assessment, Oakland, California, USA; 4 Southwest Institute for Clinical Research, Rancho Mirage, California, USA; 5 School of Nursing, University of California, Los Angeles, California, USA

**Keywords:** cardiovascular, coarse particles, epidemiology, heart rate variability, particulate matter, PM, PM_2.5_, PM_10_, PM_10–2.5_

## Abstract

Alterations in cardiac autonomic control, assessed by changes in heart rate variability (HRV), provide one plausible mechanistic explanation for consistent associations between exposure to airborne particulate matter (PM) and increased risks of cardiovascular mortality. Decreased HRV has been linked with exposures to PM_10_ (PM with aerodynamic diameter ≤ 10 μm) and with fine particles (PM with aerodynamic diameter ≤ 2.5 μm) originating primarily from combustion sources. However, little is known about the relationship between HRV and coarse particles [PM with aerodynamic diameter 10–2.5 μm (PM_10–2.5_)], which typically result from entrainment of dust and soil or from mechanical abrasive processes in industry and transportation. We measured several HRV variables in 19 nonsmoking older adults with coronary artery disease residing in the Coachella Valley, California, a desert resort and retirement area in which ambient PM_10_ consists predominantly of PM_10–2.5_. Study subjects wore Holter monitors for 24 hr once per week for up to 12 weeks during spring 2000. Pollutant concentrations were assessed at nearby fixed-site monitors. We used mixed models that controlled for individual-specific effects to examine relationships between air pollutants and several HRV metrics. Decrements in several measures of HRV were consistently associated with both PM_10_ and PM_10–2.5_; however, there was little relationship of HRV variables with PM_2.5_ concentrations. The magnitude of the associations (~ 1–4% decrease in HRV per 10-μg/m^3^ increase in PM_10_ or PM_10–2.5_) was comparable with those observed in several other studies of PM. Elevated levels of ambient PM_10–2.5_ may adversely affect HRV in older subjects with coronary artery disease.

Many epidemiologic studies have demonstrated consistent associations between exposure to airborne particulate matter (PM) and increased risks of morbidity and mortality [Bell et al. 2004; U.S. Environmental Protection Agency (EPA) 2004]. Risks of premature mortality appear to be greatest among older adults with preexisting cardiac and respiratory conditions, especially ischemic heart disease and chronic obstructive pulmonary disease (COPD). However, underlying pathophysiologic mechanisms are still unknown. It is plausible that PM-associated mortality can be explained, at least in part, by alterations in cardiac autonomic balance, as measured by heart rate variability (HRV).

HRV describes changes in consecutive sinus R-R intervals or in instantaneous heart rates recorded on an electrocardiogram (ECG) and has been associated with all-cause mortality (Tsuji et al. 1994), sudden cardiac death (Algra et al. 1993), and death due to heart failure (Szabo et al. 1997). HRV is decreased in survivors of acute myocardial infarction compared with healthy subjects (Bigger et al. 1995) and is altered in smokers (Hayano et al. 1990) and in individuals with COPD (Pagani et al. 1996). Decreased HRV has also been linked with conditions involving autonomic nervous system dysfunction, such as diabetes (Pfeifer et al. 1982) and Parkinson disease (Kuroiwa et al. 1983).

Several studies have linked exposure to ambient PM with decreased HRV (Creason et al. 2001; Gold et al. 2000; Holguin et al. 2003; Liao et al. 1999; Park et al. 2005; Pope et al. 1999, 2004; Schwartz et al. 2005). These investigations were conducted in areas where the mass of PM with aerodynamic diameter ≤ 10 μm (PM_10_) was composed primarily of fine particles [PM with aerodynamic diameter ≤ 2.5 μm (PM_2.5_)], which typically originate in combustion and photo-chemical processes. In contrast, coarse particles [PM with aerodynamic diameter between 2.5 and 10 μm (PM_10–2.5_)] are primarily derived from soil and from abrasive mechanical processes in transportation and industry (U.S. EPA 2004). At least two studies found no relationship between PM_10–2.5_ and changes in HRV (Gold et al. 2000; Liao et al. 1999); however, those investigations took place in urban areas with low PM_10–2.5_ levels. In contrast, our study is the first to examine the impact of PM on HRV in an area where PM_10–2.5_ predominates.

We previously identified associations between daily PM_10_ concentrations and cardiovascular mortality in Coachella Valley, a desert resort and retirement area east of Los Angeles, California (Ostro et al. 1999, 2000). Within this valley, widespread gusty winds occur in conjunction with large pressure gradients resulting from differences between the desert and coastal air masses, generating copious quantities of windblown sand and dust. Most of the variability in PM_10_ in the valley is attributable to PM_10–2.5_, even on days without wind events. Based on concurrent PM_10_ and PM_2.5_ fixed-site monitoring during a 2.5-year period, PM_10–2.5_ was highly correlated with PM_10_ on a daily basis (*r* ~ 0.95) (Ostro et al. 2000). Chemical mass balance modeling undertaken by the South Coast Air Quality Management District (SCAQMD 1990) indicated that geologic sources contribute approximately 50–60% of PM_10_ on an annual basis and up to 95% during wind events. In the present study, we examined whether ambient PM_10_, PM_2.5_, and PM_10–2.5_ levels were associated with changes in HRV in older adults with coronary artery disease.

## Materials and Methods

### Subject recruitment

The study protocol was approved by the Institutional Review Board of the Public Health Institute (Oakland, California). Study participants were recruited from a large cardiology practice and through newspaper advertisements from December 1999 through February 2000. Subjects were eligible if they were ambulatory adults ≥ 60 years of age; were not current smokers; had coronary artery disease manifested by at least one of the following: *a*) a history of angina and a positive ECG, echocardiographic or nuclear stress test, or angiography (*n* = 12), *b*) prior percutaneous coronary intervention (*n* = 1), *c*) prior coronary artery bypass surgery (*n* = 12), or *d*) a history of myocardial infarction at least 6 months before recruitment (*n* = 16) (of the 19 subjects in the study, almost all met at least two criteria for eligibility); and residence within 5 miles of either of the two fixed-site air quality monitoring stations in Coachella Valley (located in Palm Springs and Indio).

Exclusion criteria included conditions associated with autonomic dysfunction (e.g., diabetes, chronic renal failure, Parkinsonism, and chronic alcohol abuse), cardiac transplant, cardiac pacemaker, implantable defibrillator, atrial fibrillation, or significant cognitive impairment.

### Data collection

During the initial in-person appointment, staff obtained written informed consent and administered a baseline questionnaire, which included questions on subject demographics, medical history, current medications, usual daily activities, and any limitations on activity. The information obtained in the baseline questionnaire was supplemented by abstracting photocopies of the individuals’ medical records on standardized forms. Data abstracted from the medical records included, where available, left ventricular ejection fraction (LVEF), history of myocardial infarction, and medications prescribed. During this study, the participants remained under the medical supervision of their regular personal physicians.

Staff also measured the subjects’ lung function at baseline using a portable Simplicity spirometer (Mallinckrodt, Inc., St. Louis, MO). Spirometry was conducted following the guidelines of the American Thoracic Society (1995), with reproducibility criteria modified slightly to accommodate the subjects’ ages (i.e., results of at least three of the forced expiratory maneuvers were required to be within 15% of one another). Briefly, the subjects were seated and wore a nose clip for the spirometric maneuvers. Each subject performed at least four expiratory maneuvers. Spirometry was rescheduled for subjects who reported a respiratory infection in the preceding 3 weeks.

Twenty-four–hour ambulatory ECGs were digitally recorded for each subject at weekly intervals from 14 February through 31 May 2000, using lightweight Trillium 3000 Holter monitors with disposable electrodes (Forest Medical, Syracuse, NY). During the Holter monitoring, subjects performed their normal daily activities, except those that would interfere with the ECG recording, such as showering. In general, Holter monitoring began at the same time and day every week for each subject. In cases of missed appointments, subjects were rescheduled for monitoring within the next 2 days, if possible.

Staff followed a standardized protocol for subject preparation and placed five electrodes (two channels) in a modified V5 and aVF configuration similar to that used by Pope et al. (1999, 2004). Each Holter monitoring session began with a 20-min resting ECG with the subject supine, during which staff remained with the subject. At each session, staff gave the subject a simple 24-hr time–activity diary to record times spent indoors or outdoors, air conditioner (AC) use (yes or no), and whether windows were open during each 2-hr period (and one 6-hr block from 2400 hr to 0600 hr).

Staff downloaded each subject’s monitoring data from a removable flashcard to a personal computer for storage and subsequent editing by an ECG technician. The subjects’ physicians were sent a standard Holter report within 24 hr, which resulted in the identification of three subjects during the initial monitoring sessions who had experienced asymptomatic but potentially life-threatening arrhythmias. These patients underwent procedures to implant defibrillator/pacemaker devices and were dropped from the study; no additional recordings were undertaken for these three individuals. An additional subject was found to have continuous atrial fibrillation. None of these subjects’ ECG data were included in the analysis. Thus, of the initial 23 subjects, we had multiple ECG recordings from 19 for the analysis.

Ambient pollutant data consisted of continuous measurements of PM_10_, PM_2.5_, and ozone, which were monitored at fixed-site stations operated by the SCAQMD in Indio and Palm Springs, located at either end of the population corridor in Coachella Valley. PM_10–2.5_ data were derived by subtracting PM_2.5_ mass concentrations from PM_10_. Although the SCAQMD also monitored for carbon monoxide and nitrogen dioxide during the study period, we did not use these data for our analysis because there were many days with missing values. Sulfur dioxide was not monitored in the valley at that time. We obtained daily meteorologic data collected at two valley airports (minimum, maximum, and mean temperature, as well as dew point, relative humidity, and barometric pressure) from the National Climatic Data Center (Asheville, NC).

Data from baseline questionnaires, medical records abstraction, pulmonary function testing, daily diaries, and extracted HRV variables were entered into a SAS database, with 10% double-data entry to check for accuracy. The database was then merged with air quality and meteorologic data for analysis using SAS (version 8; SAS Institute Inc., Cary, NC).

### Data analysis

Only normal sinus R-R intervals were used in the HRV analysis. Artifacts, ectopy (both supraventricular and ventricular), and uninterpretable complexes were excluded. We examined time-domain, frequency-domain, and geometric HRV variables. Time-domain variables included *a*) the standard deviation of normal sinus rhythm (“normal-to-normal” or N-N) beats (SDNN), representing the average of the standard deviations of normal beats of successive 5-min blocks over the duration of the monitoring period (SDNN estimates overall HRV); *b*) the standard deviation of the average N-N intervals (SDANN) within successive 5-min blocks (an estimate of long-term components of HRV); and *c*) the root mean square of successive differences (r-MSSD), which is the square root of the mean of the sum of the squares of differences between adjacent normal R-R intervals, which estimates short-term components of HRV and is a sensitive indicator of vagal tone (Task Force 1996).

Frequency-domain analysis delineates the heart rate signal into its frequency components and quantifies them in terms of their relative intensity or power. We examined three frequency-domain variables: high frequency (HF), low frequency (LF), and total power. HF components (0.15–0.40 Hz) provide an index of parasympathetic activity, whereas LF components (0.04–0.15 Hz) are considered to encompass both sympathetic and parasympathetic activity (Cerutti et al. 1995; Task Force 1996). Total power is an indicator of overall HRV.

Geometric methods involve analysis of the sample density histogram of R-R interval durations. A plot of the distribution typically depicts the main peak as a triangular shape. The triangular index (TRII) provides an estimate of overall HRV that is more resistant to beat-labeling errors than are its time- and frequency-domain counterparts (Task Force 1996).

Because the Holter software did not allow for downloading time-domain HRV variables for monitoring periods other than the full 24 hr, we chose specific time intervals for which the HRV variables would be calculated and then extracted them manually into the database. For time-domain variables and the TRII, we chose 0600–0800 hr, 1800–2000 hr, and 24 hr. The two 2-hr intervals were selected to provide the most marked contrasts in ambient PM levels, which tended to be lowest in the early morning and highest in the early evening, based on examination of 24-hr continuous monitoring data from Palm Springs and Indio.

Frequency-domain variables generally are measured in 5-min increments and are sensitive to physical activity patterns. Therefore, to reduce intersubject behavioral variability, we chose to examine two 5-min periods: *a*) minutes 6–10 of the Holter monitoring session, during which the subjects were supine after the hook-up; and *b*) 0301–0305 hr, when most individuals would be asleep.

In summary, for each individual monitoring day, we obtained SDNN, SDANN, r-MSSD, and TRII for the full 24-hr period and for two 2-hr periods in the morning and evening, as well as frequency-domain HRV variables for two 5-min intervals.

### Statistical methods

Most of the HRV variables were log-normally distributed and were log-transformed for the analyses. We applied mixed linear regression models to the continuous HRV variables and pollution metrics, with random-effects parameters to control for interindividual variation and fixed-effects parameters to estimate relationships between the various pollutant metrics and changes in HRV.

We explored the independent influence of meteorologic factors, examining both concurrent and lagged values (up to 3 days) of temperature, humidity, dew point, and barometric pressure. Because only barometric pressure was associated with HRV metrics, it was retained in subsequent models. Air pollutant variables were entered individually into the models; we examined the impact of both concurrent and lagged pollutant values to allow for the possibility of delayed and cumulative effects. Therefore, for the 24-hr measures of HRV, single-day lags and moving averages of up to 4 previous days for each pollutant were considered. For HRV variables measured on a 2-hr (time domain and TRII) or 5-min (frequency domain) basis, we examined 2-, 4-, 6-, 8-, and 24-hr pollutant moving averages. Because HRV is related inversely to heart rate, the models included the subjects’ average heart rate during the monitoring periods.

For some of the associations found, we conducted additional analyses to examine potential impacts of behavioral factors that might influence exposure. For example, for the 2-hr evening period, we examined the effect (based on responses in the daily diary) of subjects’ keeping windows open, using AC, or being outdoors for > 1 hr. Each of these factors was included separately as a dichotomous variable in models that also included a PM metric (PM_10_, PM_10–2.5_, or PM_2.5_). We also added an interaction term between the specific factor and the PM metric to these models (e.g., the use of AC between 1800 and 2000 hr and concurrent PM_10_).

## Results

Table 1 presents demographic and medical data for the 19 participants. The average number of HRV monitoring sessions per subject was 8.8 (range, 4–12). Descriptive statistics for the pollutant and meteorologic variables during the study period are presented in Table 2, and the time- and frequency-domain HRV variables used in the analysis are summarized in Table 3.

Evaluation of potential time-variant confounders through both simple correlation analysis and univariate regressions indicated that the pollutant variables were not confounded by any meteorologic variables. Although barometric pressure was often associated with the HRV measures, it had little impact on the associations of ambient pollutants with HRV. Therefore, the results presented are from fixed-effects models that included only the pollutant term and average heart rate as predictor variables.

Results of the analysis of time-domain HRV variables measured during the evening period (1800–2000 hr) are displayed in Table 4. These results indicated associations between decrements in SDNN, SDANN, and TRII in relation to increases in both PM_10_ and PM_10–2.5_. The magnitude of the associations between SDNN and PM_10_ or PM_10–2.5_ increased as the averaging time increased up to 6 hr but began to decrease at 8 hr and diminished to nonsignificance when the averaging time was extended to the prior 24 hr. A similar pattern was observed for SDANN, whereas for TRII the coefficients for both PM_10_ and PM_10–2.5_ continued to increase modestly at 8 hr relative to an averaging time of 6 hr. There was no evidence of an association between PM_2.5_ or ozone and these HRV variables. There was no association between any pollutant variable and r-MSSD, except for a marginally significant but positive association with PM_10–2.5_ averaged over the preceding 24 hr.

In contrast to the regressions for the evening monitoring period, there were few associations during the morning monitoring period between pollutant metrics and time-domain variables (data not shown). PM_10–2.5_ was associated with both SDNN and SDANN at lags up to 4 hr but not at 24 hr. PM_10_, PM_2.5_, and ozone were not associated with any HRV metrics in the morning session. In addition, there was again a marginally significant positive association between PM_10–2.5_ averaged over 24 hr and r-MSSD.

Analysis of the frequency-domain variables during sleep (0300 hr) also indicated sporadic associations between HRV and PM metrics (Table 5). For this monitoring period, the unlagged pollutant variables were measured over the prior hour (i.e., 0200–0300 hr). Total power was associated with all three particulate metrics. The strongest associations for PM_10_ and PM_10–2.5_ were averaged over the prior 4 hr, whereas for PM_2.5_, only the measurement in the prior hour was statistically significant. There were also several modest associations with changes in the HF and LF components, with no obvious patterns. Ozone was also associated with decreases in all three frequency-domain measures, although the coefficients were of borderline significance (*p* = 0.08 to 0.10). The daytime posthookup frequency-domain variables also showed no pattern of association with the pollutant metrics (data not shown).

Adding variables representing exposure-related behaviors (e.g., use of AC) to the models generally resulted in modest increases in the magnitude and significance of the coefficients for PM_10_ and PM_10–2.5_ (data not shown). However, neither these behavioral variables nor the interactive term coefficients were statistically associated with the HRV metrics. The use of exposure adjustment factors did not alter the generally null to modest findings for PM_2.5_.

Several constitutional and clinical variables [age, sex, lung function, use of beta-blockers or angiotensin-converting enzyme (ACE) inhibitors, prior smoking status] did not exhibit an association with SDNN, nor did they have much, if any, effect on the magnitude or significance of PM_10–2.5_ coefficients. In contrast, inclusion of LVEF in the model increased the absolute magnitude of the PM_10–2.5_ coefficient by 36% [from −0.00072 (*p* = 0.02) to −0.00098 (*p* = 0.007)], whereas the LVEF coefficient was of borderline statistical significance (*p* = 0.09).

## Discussion

We found consistent associations of several PM metrics, notably PM_10_ and PM_10–2.5_, with short-term decrements in several measures of HRV in a panel of older adults with coronary artery disease. The strongest associations were detected when PM measurements were taken within a few hours before the HRV measures. These associations, however, were no longer present when the PM averaging time was extended to 24 hr or longer. These observations suggest that if there are causal relationships between PM exposures and decreases in HRV, the effects likely occur in close temporal proximity to the exposures.

These findings accord with some previous epidemiologic studies of HRV (Gold et al. 2000; Pope et al. 2001), although others have reported more prolonged effects (Creason et al. 2001; Pope et al. 2004). Gold et al. (2000) conducted 25-min ECG measurements in 21 older Boston residents weekly over a 3-month period. They reported significant associations of r-MSSD and SDNN with PM_2.5_ within a few hours of obtaining the ECG data. No associations between PM_2.5_ and HRV were seen at lags longer than 24 hr. In a subsequent study, however, the same researchers found somewhat stronger associations with 24-hr PM metrics than with 4-hr averages (Schwartz et al. 2005). Pope et al. (2004) reported decrements in several HRV metrics associated with 24-hr averages of PM_2.5_ measured up to 2 days before Holter monitoring, although the strongest associations were with same-day measurements. A recent study of 10 elderly subjects involving 2-hr controlled exposures to either filtered air or concentrated PM_2.5_ also reported significant decrements in several HRV measures immediately postexposure, which tended to persist (albeit somewhat attenuated) at 24 hr postexposure (Devlin et al. 2003). In contrast, other investigators found that a 48-hr PM averaging time had the strongest associations with decrements in HRV (Park et al. 2005).

In our study population of individuals with coronary artery disease, we identified PM-associated decreases in SDNN, SDANN, and TRII, but little relationship with r-MSSD. Others have found decrements in SDNN and SDANN, with mixed results regarding r-MSSD (Pope et al. 1999, 2004). It is possible that the variable results with the latter metric are caused partly by the effects of a variety of common cardiovascular medications on r-MSSD. Liao et al. (1999) examined HRV in 26 elderly residents of a Baltimore retirement home, reporting significant decreases in HF, LF, and SDNN in relation to indoor and outdoor PM_2.5_ only among subjects with preexisting cardiovascular disease. Recently, Schwartz et al. (2005) reported stronger associations of PM_2.5_ (especially black carbon) with HRV decrements in subjects with a prior myocardial infarction (*n* = 3) relative to the other subjects (*n* = 25), although this observation must be interpreted cautiously because of small numbers. Other studies have reported that subjects with cardiovascular disease may be at increased risk of PM-associated changes in HRV (Holguin et al. 2003; Park et al. 2005). Holguin et al. (2003) reported decrements in HF and LF variables among 34 elderly nursing home residents with both PM_2.5_ and ozone in Mexico City, especially among individuals with hypertension. However, we found little relationship between frequency-domain variables and either of these pollutants, nor did we observe that a history of hypertension affected the PM–HRV associations. However, the levels of both PM_2.5_ and ozone were substantially greater in the Mexican study (means of 37.2 μg/m^3^ PM_2.5_ and 149 ppb ozone in Mexico City vs. 18.6 μg/m^3^ and 37 ppb, respectively, in our study, representing the averages of the values recorded at Indio and Palm Springs). In addition, all of the subjects in our study had documented coronary artery disease, which could represent a more important determinant of susceptibility compared with hypertension alone.

Direct comparisons of our quantitative results with those of prior air pollution–HRV investigations are not entirely appropriate because they involve multiple differences in study design (e.g., various pollutant mixtures, averaging times for both pollution and HRV, exposure scenarios, subjects’ health status and medications, and Holter monitoring protocols). Nevertheless, our results suggest associations of HRV metrics with PM exposures of the same order of magnitude as several of those previously reported (Table 6), although not all studies have identified a relationship between PM and HRV.

In contrast to prior studies, however, the strongest signals that we identified were associated with PM_10–2.5_ of primarily geologic origins, which dominate the PM_10_ mass in the Coachella Valley, as well as throughout much of the arid American West and Southwest. A chemical mass balance analysis of annual average particle composition in Indio conducted previously by the SCAQMD (1990) indicated that geologic and vehicular sources contributed approximately 59% and 8% of PM_10_ mass, respectively, with high particulate metal concentrations of silicon, aluminum, iron, and calcium, markers of crustal sources. That we found associations of PM_10–2.5_ with HRV decrements whereas others (e.g., Liao et al. 1999) did not may be a dose-related phenomenon: PM_10–2.5_ levels were substantially higher in this study than in any of the other investigations. Even within our study, we identified stronger, more consistent associations with the evening than with the morning PM concentrations (mean PM_10–2.5_ levels of 47.1 μg/m^3^ and 18.3 μg/m^3^ for the 2-hr evening and morning periods, respectively), suggesting a concentration-related effect. In addition, PM_10–2.5_ composition in the valley likely differs from those found in urban settings. It is also possible that at least some of the particles of interest lie in the intermodal size range (where the PM_2.5_ and PM_10–2.5_ distributions overlap).

Several publications link ambient PM_10–2.5_ with cardiovascular mortality (Castillejos et al. 2000; Mar et al. 2000; Ostro et al. 2000) and morbidity (Tolbert et al. 2000). A recent review of the limited epidemiologic evidence found support for associations between PM_10–2.5_ and cardiorespiratory morbidity and mortality (Brunekreef and Forsberg 2005). Toxicologic studies also indicate that some PM_10–2.5_ may be at least as capable of eliciting proinflammatory effects and oxidative damage as PM_2.5_ (Monn et al. 1999; Schins et al. 2004). Although particles may initiate or enhance inflammatory processes in the airways, the HRV changes indicated by our results and those of others (Gold et al. 2000; Pope et al. 2001) occurred over a time course shorter than that typically linked with pollutant-induced inflammation, suggesting perhaps direct involvement of neural reflexes. Consistent with this observation are the results of a recent controlled exposure study of asthmatic and healthy young adults (Gong et al. 2004), in which concentrated PM_10–2.5_ exposures resulted in modest decrements in HRV with no evidence of airway inflammation in induced sputum or changes in exhaled nitric oxide. Using a PM_10–2.5_ concentrator, Gong et al. (2004) found reductions in several HRV measures in four healthy adults exposed for 2 hr to concentrations ranging from 46 to 197 μg/m^3^. Although the sample size was quite small, that investigation provides a modest degree of corroboration of our findings.

That we found sporadic and relatively weaker associations of HRV decrements with PM_2.5_ is somewhat puzzling, because the ambient PM_2.5_ concentrations in our study over-lapped with those in several other investigations (Gold et al. 2000; Liao et al. 1999). However, the specific composition and sources of the particles may be important determinants of response. Schwartz et al. (2005) found that the black carbon fraction of PM_2.5_ showed a stronger relationship with decrements in frequency- and time-domain HRV variables than did secondary particles (e.g., sulfates), although the latter were not measured directly in that analysis. In contrast, in a study of 34 elderly subjects in Seattle, Washington, where residential wood combustion is an important source of PM_2.5_, Sullivan et al. (2005) found no association of several frequency-domain HRV variables with 1-hr, 4-hr, or 24-hr measurements of ambient or indoor PM_2.5_. However, in that study, concentrations were low relative to those in other epidemiologic studies, and there was limited variability in exposure (median interquartile range was 6 μg/m^3^); thus, the extent to which wood combustion or other sources might have influenced these results cannot be determined. In a study of 39 boiler-makers exposed to PM_2.5_ occupationally, Magari et al. (2002) reported significant associations of PM-associated vanadium and lead with increases in the SDNN index after adjusting for mean heart rate, age, and smoking status, suggesting that specific particulate metals may affect cardiac autonomic activity.

Having detected consistent associations between PM metrics and several HRV variables in this population of individuals with preexisting cardiovascular disease, we did not identify any subject characteristics, except LVEF, associated with heightened susceptibility to PM. However, with only 19 subjects, this secondary analysis can only be considered exploratory. Moreover, LVEF was strongly correlated with average heart rate (*r* = 0.46, *p* < 0.0001), which is inversely related to HRV.

## Conclusions

In this study of elderly subjects with documented coronary artery disease, we detected decrements in several HRV metrics that were consistently associated with elevated PM_10_ and PM_10–2.5_ concentrations. Associations of these HRV variables with PM_2.5_ levels were generally much weaker. The magnitude of the associations (~1–4% decrease in HRV metrics per 10-μg/m^3^ increase in PM_10_ or PM_10–2.5_) was comparable with those observed in other studies of PM_2.5_ in urban areas. In arid environments characteristic of much of the American West and Southwest, elevated levels of ambient PM_10–2.5_ may adversely affect HRV in older subjects with coronary artery disease.

The clinical significance of PM-associated HRV decrements remains to be established. Most studies linking decreases in HRV with increased mortality have examined 24-hr baseline HRV as a predictor of adverse events over the course of months to years. Although these and others’ results may offer a partial mechanistic explanation for the repeated observations in time-series studies of PM-associated cardiovascular mortality, it is also possible that such acute decrements in HRV represent an epiphenomenon of some other unmeasured, underlying pathophysiologic processes.

## Figures and Tables

**Table 1:** Characteristics of HRV study population (*n* = 19).

Characteristic	Value
Age (years)	71.3 ± 6.0
Sex (no.)	
Male	12
Female	7
Smoking status (no.)	
Never	7
Former	12
Cardiac medications (no.)	
Beta blockers	9
ACE inhibitors	7
Calcium channel blockers	2
Lung function (mean ± SD)	
FEV_1_ (L)	2.17 ± 0.58
FVC (L)	3.01 ± 0.85
FEF_25–75%_ (L/sec)	1.83 ± 0.98
FEV_1_/FVC	0.77 ± 0.02
FEF_25–75%_/FVC	0.64 ± 0.37
LVEF (mean ± SD)	47% ± 17

Abbreviations: FEF_25–75%_, mean forced expiratory flow between 25% and 75% of the FVC; FEV_1_, forced expiratory volume at 1 sec; FVC, forced vital capacity; LVEF, left ventricular ejection fraction.

**Table 2:** Descriptive statistics of pollutant and meteorologic variables.

Pollutant or meteorologic variable	Mean (range)
PM_10_ (μg/m^3^, 24-hr average)	
Indio	46.1 (11.8–289.2)
Palm Springs	31.0 (9.0–140.3)
PM_2.5_ (μg/m^3^, 24-hr average)	
Indio	23.2 (6.3–90.4)
Palm Springs	14 (4.7–52)
Ozone (ppb, 1-hr maximum)	
Palm Springs	41 (19–92)
Indio	33 (12–66)
Maximum temperature (°F)	69.6 (49.8–88.4)
Relative humidity (%)	44.2 (13.7–85.5)
Barometric pressure (mb)	29.4 (29.2–29.7)
Precipitation (in)	0.0006 (0–0.010)

**Table 3:** Summary of HRV variables^a^ and average heart rate.

Variable (unit)	No.	Mean ± SD
SDNN (msec)	168	73.2 ± 29.3
SDANN (msec)	168	53.9 ± 22.8
R-MSSD (msec)	168	44.1 ± 42.5
TRII	168	17.2 ± 6.1
Total power (msec^2^)	169	1,216 ± 1,915
HF (msec^2^)	169	304 ± 818
LF (msec^2^)	169	221 ± 332
Average heart rate (beats/min)	168	76 ± 13

a Time domain variables, TRII, and average heart rate were measured 1800–2000 hr. Frequency domain variables were measured at 0300 hr.

**Table 4:** Regression coefficients^a^ for time-domain HRV variables measured in the evening (1800–2000 hr) in relation to different averaging times for PM_10_, PM_10–2.5_, and PM_2.5_.

	SDNN		SDANN		TRII	
PM moving average	Coefficient (SE)	*p*-Value	Coefficient (SE)	*p*-Value	Coefficient (SE)	*p*-Value
PM_10_						
1800–2000 hr	−0.71 (0.268)	0.009	−0.99 (0.366)	0.008	−0.72 (0.252)	0.005
1600–2000 hr	−1.03 (0.433)	0.019	−1.38 (0.590)	0.021	−1.1 (0.406)	0.008
1400–2000 hr	−1.45 (0.54)	0.008	−1.80 (0.738)	0.016	−1.41 (0.51)	0.007
1200–2000 hr	−1.31 (0.60)	0.031	−1.53 (0.821)	0.064	−1.46 (0.563)	0.011
24 hr	−0.51 (0.77)	0.510	−0.71 (1.072)	0.51	−1.00 (0.725)	0.17
PM_10–2.5_						
1800–2000 hr	−0.72 (0.296)	0.017	−0.96 (0.444)	0.034	−0.61 (0.303)	0.046
1600–2000 hr	−1.19 (0.516)	0.024	−1.53 (0.765)	0.049	−0.89 (0.531)	0.096
1400–2000 hr	−1.84 (0.649)	0.006	−2.37 (0.986)	0.019	−1.23 (0.691)	0.080
1200–2000 hr	−1.4 (0.767)	0.074	−2.02 (1.159)	0.087	−1.62 (0.833)	0.056
24 hr	0.23 (0.923)	0.81	0.42 (1.433)	0.77	−0.37 (1.148)	0.75
PM_2.5_						
1800–2000 hr	−0.37 (1.01)	0.72	−1.26 (1.375)	0.36	−0.76 (0.957)	0.43
1600–2000 hr	−0.55 (1.176)	0.64	−1.66 (1.60)	0.30	−0.55 (1.087)	0.61
1400–2000 hr	−1.21 (1.034)	0.24	−1.74 (1.391)	0.21	−0.43 (0.956)	0.65
1200–2000 hr	−1.25 (1.122)	0.27	−1.54 (1.429)	0.28	−0.26 (1.018)	0.80
24 hr	−1.63 (2.36)	0.49	−3.20 (3.153)	0.31	−0.96 (2.129)	0.65

a All coefficients and SE × 1,000. Regression model includes pollutant variable and average heart rate. Coefficient represents relationship between exposure and ln(SDNN) in msec.

**Table 5:** Regression coefficients for frequency-domain HRV variables in relation to PM metrics and ozone.

	Total power		HF		LF	
Pollutant^a^	Coefficient (SE)	*p*-Value	Coefficient (SE)	*p*-Value	Coefficient (SE)	*p*-Value
PM_10_	−4.48 (2.732)	0.13	0.593 (2.829)	0.83	−3.40 (2.493)	0.17
2 hr	−6.35 (3.479)	0.07	−0.12 (3.612)	0.98	−4.66 (3.177)	0.15
4 hr	−8.10 (3.546)	0.02	−1.62 (3.684)	0.66	−5.65 (3.239)	0.08
24 hr	−1.14 (3.192)	0.72	−4.02 (3.156)	0.21	0.293 (3.004)	0.92
PM_2.5_	−11.00 (5.017)	0.03	3.112 (5.252)	0.55	−7.90 (4.642)	0.09
2 hr	−7.28 (4.588)	0.12	3.884 (4.757)	0.42	−5.28 (4.249)	0.22
4 hr	−6.45 (4.619)	0.17	5.111 (4.774)	0.29	−4.78 (4.263)	0.27
24 hr	−1.84 (7.733)	0.81	15.84 (8.480)	0.07	0.794 (7.403)	0.92
PM_10–2.5_	−4.61 (3.533)	0.20	−0.36 (3.469)	0.92	−3.38 (3.202)	0.29
2 hr	−6.16 (5.109)	0.23	1.794 (5.077)	0.73	−4.05 (4.795)	0.40
4 hr	−13.60 (6.438)	0.04	−3.51 (6.244)	0.58	−8.69 (5.964)	0.15
24 hr	−1.02 (4.824)	0.84	−4.04 (4.483)	0.38	−1.37 (4.213)	0.75
Ozone	−82.80 (63.03)	0.19	−106.60 (62.09)	0.09	−77.92 (58.73)	0.19
8 hr	−147.40 (88.37)	0.10	−45.52 (91.09)	0.62	−145.70 (82.15)	0.08

a All coefficients and SE × 1,000. PM_10_ indicates PM_10_ levels measured in the hour just before the HRV measurement (0300 hr); PM_10_ 2 hr indicates PM_10_ levels measured in the 2 hr before the HRV measurement, and so forth. Similar conventions apply to PM_2.5_, PM_10–2.5_, and ozone, except that ozone 8-hr indicates 8-hr averaged ozone levels (1900 hr–0300 hr).

**Table 6:** Comparisons of particle-associated decreases in SDNN per 10 μg/m^3^ increase in PM.

Reference	Mean of SDNN (averaging time)	PM metric (averaging time)	Effect estimate [msec (95% CI)]	Percent change
Pope et al. 1999	129.6 (24 hr)	PM_10_ (24 hr)	−1.8 (−1.1 to −2.5)	−1.4
Gold et al. 2000	72.7 (25 min)	PM_2.5_ (4 hr)	−2.6 (−0.8 to −4.4)	−3.6
Pope et al. 2001	74.7 (1.75 hr)	RSP (1.75 hr)	−1.1 (−0.5 to −1.7)	−1.5
Pope et al. 2004	131.4 (24 hr)	PM_2.5_ (24 hr)	−3.5 (−1.9 to −5.1)	−2.7
Park et al. 2005	31.6 (4 min)	PM_2.5_ (24 hr)	−0.9 (−3.0 to 1.4)	−2.8
Sullivan et al. 2005	49 (20 min)	PM_2.5_ (24 hr)	0.5 (−2.4 to 3.9)^a^	1.0
Chuang et al. 2005	33.9 (16 hr)	PM_2.5–1_ (4 hr)	−1.4 (−3.0 to 0.2)^a^	−4.2
Present study	73.2 (2 hr)	PM_10_ (2 hr)	−1.2 (−0.3 to −2.1)	−1.6
Present study	73.2 (2 hr)	PM_10_ (6 hr)	−2.4 (−0.7 to −4.1)	−3.3
Present study	73.2 (2 hr)	PM_10–2.5_ (2 hr)	−1.2 (−0.2 to −2.2)	−1.6
Present study	73.2 (2 hr)	PM_10–2.5_ (6 hr)	−3.0 (−1.0 to −5.1)	−4.1

Abbreviations: CI, confidence interval; RSP, respirable particles (< 3 μm) from environmental tobacco smoke.

a Subjects with cardiovascular disease.
